# Early Identification of Poorly Performing Implants in Michigan With the Example of the Vanguard XP

**DOI:** 10.1016/j.artd.2024.101478

**Published:** 2024-10-14

**Authors:** Nicholas B. Frisch, Michael A. Masini, Huiyong Zheng, Richard E. Hughes, Brian R. Hallstrom, David C. Markel

**Affiliations:** a Ascension Providence Rochester Hospital, Rochester Hills, MI, USA; b Trinity Health, IHA-Ann Arbor Orthopaedic Surgery, Ypsilanti, MI, USA; c MARCQI Coordinating Center, University of Michigan, Ann Arbor, MI, USA; d Department of Orthopaedic Surgery, University of Michigan, Ann Arbor, MI, USA; e The CORE Institute, Phoenix, AZ, USA; f Ascension Providence Hospital, Southfield, MI, USA

**Keywords:** Total knee arthroplasty, TKA, Bicruciate, Registry

## Abstract

**Background:**

Arthroplasty registries play a critical role in improving the quality of care and performing post-market surveillance of medical devices. We report the Michigan Arthroplasty Registry Collaborative Quality Initiative (MARCQI) findings specific to the Biomet Vanguard XP bicruciate-retaining total knee implant.

**Methods:**

Data were collected from MARCQI’s 2019 report (February 15, 2012, through December 31, 2018). Demographic data were analyzed to determine differences between Vanguard XP and all other implants. The cumulative percent revision (CPR) was computed from the survival function, S(t), using CPR(t) = 100∗(1 − S(t)). A log-rank test was used to assess differences in the CPR curve for the Vanguard XP and all other implants.

**Results:**

There were 148,832 knee arthroplasty cases in the MARCQI registry and 507 using Vanguard XP implant combinations. The unadjusted cumulative percent revision curve up to 5 years postoperatively for the Vanguard XP differed from all other implants (*P* < .0001). The hazard ratios for the 3 factors included in the Cox proportional hazards model were all significantly different from unity: implant (2.76, 95% CI: 1.98-3.86), sex (0.80, 95% CI: 0.74-0.85), and age (0.96, 95% CI: 0.96-0.97). The top 3 reasons for revision were pain, arthrofibrosis, and aseptic loosening. All surgeons who used the Vanguard XP experienced higher failure rates.

**Conclusions:**

The Vanguard XP experienced higher early failure rates than other TKA implants within the MARCQI registry. The development of thresholds and benchmarks for registry reporting in collaboration with industry could potentially save patients from the morbidity caused by implants that do not perform as well as anticipated.

## Introduction

Arthroplasty registries play a critical role in improving the quality of care for patients and performing postmarket surveillance of medical devices. For example, the Australian Orthopaedic Association National Joint Replacement Registry and the National Joint Registry of England, Wales and Northern Ireland identified the ASR (articular surface replacement, Depuy) metal-on-metal hip as having an unexpectedly high risk of revision before the failures were widely recognized. There was a drastic decline in utilization of this implant worldwide, largely as a result of peer-reviewed publications by these registries [1]. Without a global infrastructure for identifying outlier implants, it falls to each individual registry to report on implant performance in its annual report and/or publish in peer-reviewed literature. Moreover, novel implant designs often appear in one country or region before others. This makes it imperative that registries publish their results so other countries can develop surveillance plans.

The Biomet Vanguard XP knee system was approved for distribution through the US Food and Drug Administration 510K, substantial equivalence process in March 2013. A clinical trial of the implant was started in Denmark and is posted on clinicaltrials.gov (https://clinicaltrials.gov/ct2/show/study/NCT01966848), but no results were ever reported back to the site. One article was published by the investigators in 2020 recommending against use of the device [2]. The implant is no longer available on the Zimmer Biomet website, but there has been no recall or notification to the US Food and Drug Administration evident on the US Food and Drug Administration website. It was apparently removed from the market in 2019.

The Vanguard XP implant was used in Michigan, where the Michigan Arthroplasty Registry Collaborative Quality Initiative (MARCQI) has data collection and analysis methods in place for conducting postmarket surveillance of hip and knee arthroplasty implants [3,4]. The Vanguard XP was a bicruciate-retaining total knee replacement added as a novel option in the Vanguard total knee system. It was created to provide the flexibility to change from an anterior cruciate ligament (ACL)/posterior cruciate ligament retaining system to a posterior cruciate retaining system intraoperatively.

The ability to identify poorly performing implants in a registry is limited by the volume of cases available and the complexities of separating the implant effects from other confounding factors in the observational data. When an implant appears to have worse performance than other similar implants, the registry can potentially protect patients from further use of that implant by reporting their results. This article reports MARCQI’s findings specific to the Vanguard XP bicruciate-retaining total knee implant and explores opportunities to improve the timeliness and reliability of reporting. The results for the Vanguard XP that were reported in the 2019 MARCQI annual report are an example of the information that needs to be available to patients, surgeons, hospitals and manufacturers to improve results and reduce the need for revision in the future.

## Material and methods

This study used data from MARCQI and was exempt from institutional review board. Data collected by MARCQI were used for this analysis. MARCQI is a state-wide consortium of surgeons, hospitals, and ambulatory surgery centers dedicated to improving the quality of care for elective hip and knee arthroplasty patients (for more details, refer to the study by Hughes et al. [5]). MARCQI produces a publicly available annual report (https://marcqi.org/marcqi-registry-reports-marcqi-annual-reports) that provides surgeon volume, demographics, revision risk, number of cases at risk by year, reason for revision, type of polyethylene, approach, and utilization over time by implant product name. Implants are included in the report when there are more than 500 cases in the registry. The 2019 report covered MARCQI activities from February 15, 2012 through December 31, 2018 [6].

Demographic data were analyzed using Chi-squared and independent 2-group *t*-tests to determine if there were differences in cases between Vanguard XP and all other implants. Descriptive statistics were also tabulated. The cumulative percent revision (CPR) was computed from the survival function, S(t), using CPR(t) = 100∗(1 − S(t)). The S(t) was estimated using the Kaplan-Meier method. A log-rank test was used to assess differences in the CPR curve for the Vanguard XP and all other implants. A Cox proportional hazards model was also used to assess the impact of age and sex on the hazard function for revision. The comparative group used was all other TKA implants.

Due to a statistically significant elevated risk of revision compared to all other implants and an elevated hazard ratio for the Vanguard XP when adjusting for age and sex, additional analyses were performed. This analysis included the reasons for revision as recorded from charts reviewed by MARCQI clinical data abstractors. The first step was to analyze the hazard function over time rather than just in a Cox model. An analysis was then performed to compare reasons for revision before and after 2 years of surgery. Additional analysis was performed on the revisions performed before 1 year after surgery and between 1 and 2 years after surgery, including the causes of revision on a yearly basis thereafter. The indication for revision before and after 2 years was then analyzed relative to pain as the reason for revision. A Fisher’s exact test was used to analyze the indication for revision before and after the 2 year time point from the index procedure.

Cumulative sum (CUSUM) charts adapted for arthroplasty [7,8] were constructed for each individual surgeon in MARCQI. This tool adjusts for patient-level factors and shows risk-adjusted revision results over time and allows for investigation of time trends in revision rate for an individual surgeon. All CUSUM plots for surgeons using the Vanguard XP were scrutinized for temporal patterns.

## Results

**Figure 1:**
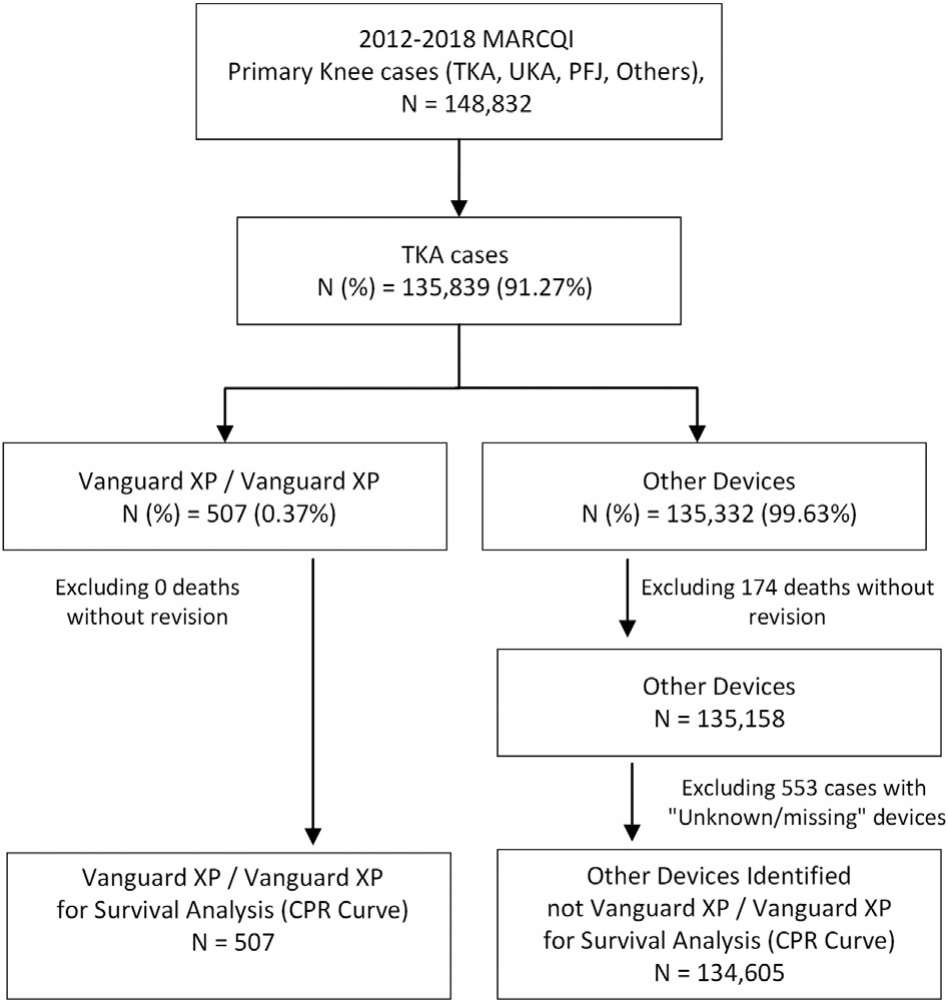
Flow diagram of cases used in analysis.

There were 148,832 knee arthroplasty cases in the MARCQI registry. When cases containing unknown/missing data and deaths were excluded, there were 507 that used a Vanguard XP implant combination and 134,605 cases that used other implants (Fig. 1). The 2 groups (Vanguard XP and all others) differed in gender, height, weight, and body mass index (Table 1), but there were no differences for smoking history or age. The mean number of Vanguard XP cases per surgeon was 39 (standard deviation 112) and the median number was 4 (interquartile range of 12). Thirteen surgeons at 13 different sites performed primary surgeries with the Vanguard XP.

**Table 1:** Descriptive statistics for Vanguard XP and all others excluding Vanguard XP.

Variables	Vanguard XP total TKA N = 507		All others excluding Vanguard XP total TKA N = 134,605		Comparisons *P* value
	N cases	Percent %	N cases	Percent %	
Sex, female, N (%)	298	58.8%	84,641	62.9%	.0009^a^
Smoking, never, N (%)	248	48.9%	69,639	51.7%	.3505^a^^NS^
Previous, N (%)	210	41.4%	51,826	38.5%	
Current, N (%)	48	9.5%	12,341	9.2%	
Unknown, N (%)	1	0.2%	799	0.6%	

	Mean (SD)		Mean (SD)		
Age (y), Mean (SD)	507	66.1 (9.1)	134,605	66.2 (9.5)	.8422^b^^NS^
Height (cm), Mean (SD)	507	169.1 (9.8)	134,594	168.0 (10.5)	.0095^b^
Weight (kg), Mean (SD)	507	87.9 (18.8)	134,594	94.3 (21.7)	<.0001^b^
BMI (kg/m^2^), Mean (SD)	507	30.8 (5.7)	134,592	33.4 (6.9)	<.0001^b^

BMI, body mass index; NS, nonsignificant at alpha = 0.05 level; SD, standard deviation.

a Chi-square *P* value for Vanguard XP and all others.

b *P* value from independent 2-group *t*-test for continuous variables between Vanguard XP and all others.

**Figure 2:**
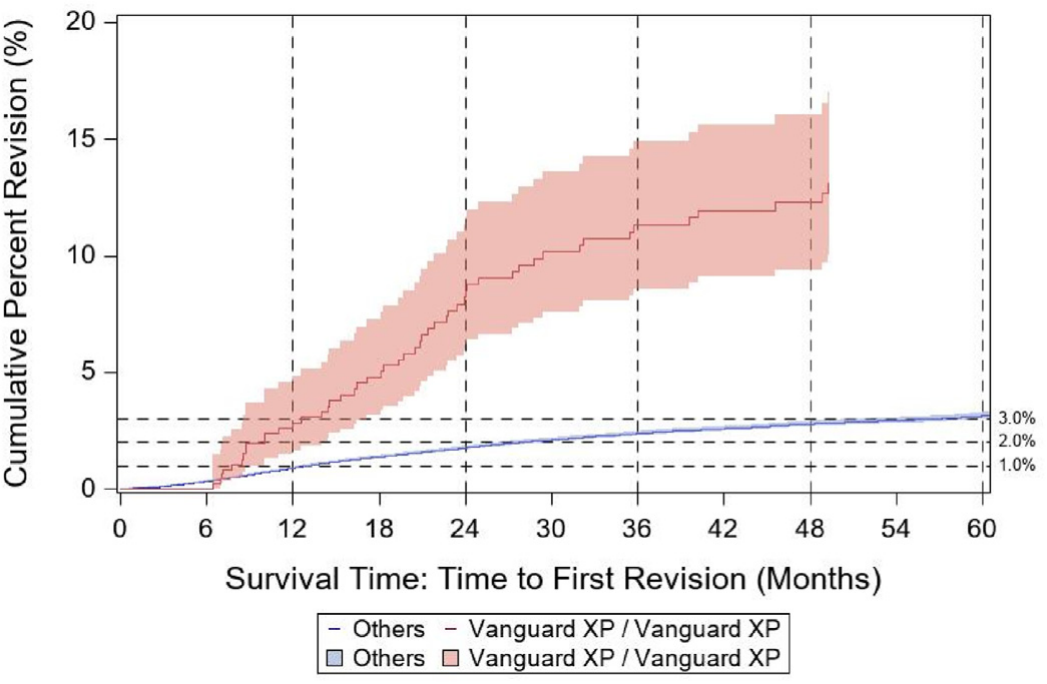
Cumulative percent revision (CPR) for the Vanguard XP to 5 years following primary procedure with shaded 95% confidence intervals.

**Figure 3:**
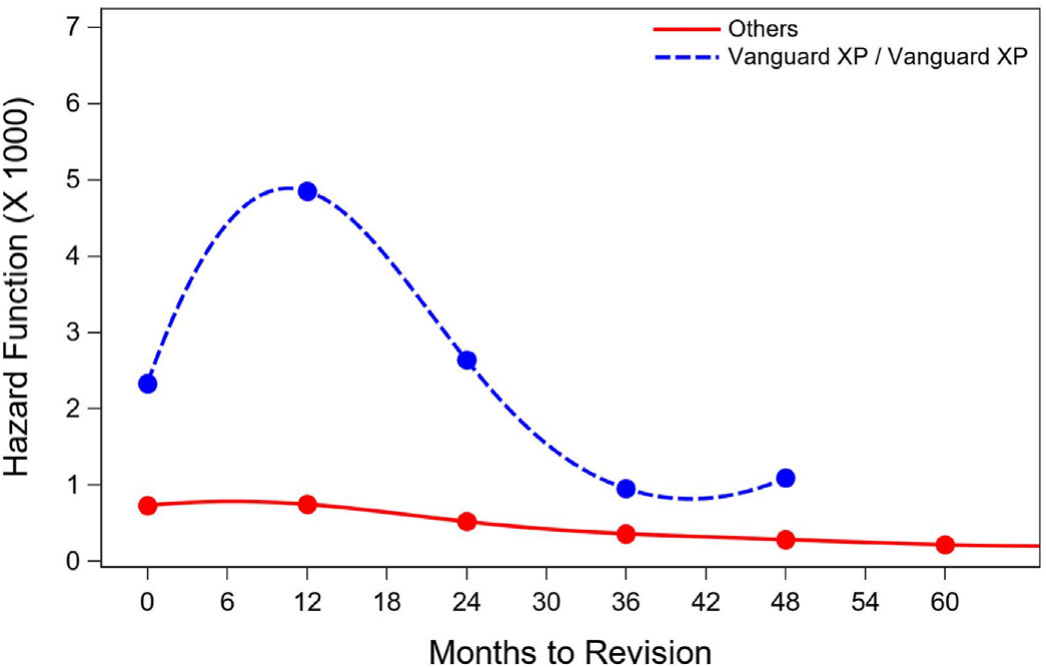
Hazard function for Vanguard XP to 5 years following primary procedure.

The Vanguard XP implant had a 9.9% revision rate at 3 years and 12.0% at 5 years. The unadjusted CPR curve up to 5 years postoperatively (Fig. 2) for the Vanguard XP differed from the CPR curve for all other implants in MARCQI (*P* < .0001). The number of cases at risk by year was 507 at time of primary surgery, 423 at 1 year, 333 at 2 years, 299 at 3 years, 226 at 4 years, and 79 at 5 years. The hazard ratios for the 3 factors included in the Cox proportional hazards model were all significantly different from unity: implant (2.76, 1.98-3.86, 95% confidence interval [CI]), sex (0.80, 0.74-0.85, 95% CI), and age (0.96, 0.96-0.97, 95% CI). The hazard function of revision (Fig. 3) increased to a peak at about 1 year postoperatively, and decreased after that time point (the hazard of revision is the instantaneous failure rate at a particular time, *t*, given that the item has already survived past time *t*).

The top 3 reasons for revision were pain, arthrofibrosis, and aseptic loosening (Table 2). This was true across hospital sites and surgeons. The most common reasons for revision in the first 2 years after surgery were pain and arthrofibrosis. The most common reasons for revision after 2 years of surgery were aseptic loosening and instability. Pain was statistically significant as a reason for revision in the first 2 years after the index procedure as compared to revision after 2 years (*P* < .05).

**Table 2:** Reason for revision for Vanguard XP implant.

Reason for revision	Overall		Revised within 2 years		Revised after 2 years	
	Frequency	Percent	Frequency	Percent	Frequency	Percent
Pain	16	36.4	14	48.3	2	13.3
Arthrofibrosis	9	20.5	6	20.7	3	20.0
Aseptic loosening	8	18.2	4	13.8	4	26.7
Dislocation/instability	7	16.0	3	10.3	4	26.7
Joint infection	3	6.8	1	3.5	2	13.3
Extensor mechanism failure	1	2.3	1	3.5	0	0

The surgeon’s effect was examined by reviewing the CUSUM charts for each surgeon using the Vanguard XP combination. This review indicated that all of the surgeons who used the Vanguard XP experienced higher failure rates than before they used the implant. After the implant was discontinued in the market, the surgeon CUSUM values returned to levels similar to those prior to use of the Vanguard XP use.

## Discussion

While most patients demonstrate improvement in pain and function after total knee arthroplasty (TKA), some patients remain dissatisfied with their results [9]. Other than infection, instability is among the most common cause of failure of both cruciate-retaining and posterior-stabilized TKA’s [10,11]. Different implant designs have emerged over the years to improve instability and increase patient satisfaction but the fact that instability remains such an issue regardless of traditional cruciate-retaining and posterior-stabilized designs makes it challenging to understand.

One commonality of most designs, regardless of implant manufacturer, is that the ACL is sacrificed. While the role of the ACL in the knee is complex, and not well understood as it relates to a TKA, some implant designs have emerged that retain the ACL with the intent of improving outcomes. The concept of bicruciate-retaining TKA predate modern TKA designs with implants such as the Polycentric, Anatomic, Geomedic, and Duocondylar [12]. Bicruciate-retaining implants were first developed in 1967 by Dr. Gunston and several different variations on the design followed [13]. In the 1980s, Cloutier, in France, designed and implanted 163 Hermes 2C “ACL-sparing” TKAs in 130 patients [14]. In Michigan, Dr. Robert Townley developed his own version of the bicruciate-retaining knee arthroplasty and used this implant extensively throughout his career and demonstrated reliable outcomes with a favorable long-term outcomes [15,16].

Potential benefits of the bicruciate-retaining design have previously been reported to include more normal knee kinematics [14,[17], [18], [19], [20], [21]], increased proprioception [[22], [23], [24], [25], [26], [27]], and potentially higher rates of patient satisfaction [[28], [29], [30]]. There are numerous studies that explore the kinematics of different TKA designs and how these altered kinematics may impact knee stability and perhaps even patient satisfaction [14,31]. In addition, Komistek et al. [18] and Halewood et al. [20] both showed similar kinematics when comparing bicruciate-retaining designs and the native knee.

Despite the theoretical benefits of the bicruciate-retaining implant design, the MARCQI Annual Report in December of 2019 raised concerns about the short-term outcomes of the Vanguard XP compared to other implant designs in the database. Prior to this, in 2017, Christensen et al. [32] reported a high early failure rate for this design with a 5% revision rate at just over 1 year and suggested further study [33]. Alnachoukati et al. [30] reported on the Vanguard XP in 2018 in a series of 146 TKAs performed between October 2014 and December 2016 by the senior author at a single institution. Follow-up was an average of 12 months (range, 1-33 months) postoperatively. Of all 146 bicruciate-retaining devices implanted, there were 2 (1.4%) revisions and 1 (0.7%) reoperation, a manipulation under anesthesia, and 9 (6.2%) knees had a minor fracture of the tibial bone island. Despite the technical difficulties, the authors concluded “the results of our study show great patient-reported satisfaction, function, and short-term outcomes for patients implanted with the new bicruciate-retaining design.” In 2019, Pelt et al. [33] followed up on the series of patients initially reported by Christensen at 1 year and found a 12% revision rate at 3 years.

In the 2019 MARCQI annual report, the Vanguard XP implant had a 9.9% revision rate at 3 years and 12.0% at 5 years, reinforcing concerns about the routine use of the implant. The CUSUM plots for revision by surgeon indicated that all of the surgeons who used the Vanguard XP experienced higher failure rates than was observed before its use and that surgeon failure rates returned to similar levels after discontinuing the implant’s use. This is a very important point to emphasize and implies that the implant itself or the complexities associated with its implantation were the reason for failure. It also suggests that there is a potential opportunity for additional education and training on newer implants. If higher complication rates are able to be identified early, the etiology could be further explored and perhaps additional technical guidance from the manufacturer or developer may provide an early solution to decrease the complication rate. Furthermore, this implant was not officially recalled, however it was voluntarily withdrawn from the market by Zimmer Biomet and to our knowledge is no longer commercially available.

Each of these results was above the thresholds established by groups attempting to classify implant outcomes. The Orthopaedic Data Evaluation Panel uses 3.5% as their criteria for designation at 3 years and 4% at 5 years [Available at: https://www.odep.org.uk/methodology/methodology-for-tkr]. The International Prosthesis Working Group benchmarking document recommends that implants have a revision rate below 2% at 2 years and 3% at 5 years [International Prosthesis Benchmarking Working Group. Guidance document: hip and knee arthroplasty devices; May, 2018. Available from: https://www.isarhome.org/publications] [3].

There are several limitations to this report. First, being a database study, the information available is limited to standardized data extraction elements. As such, more granular information surrounding the implants is not available for comment. Examples include intraoperative findings at the time of revision, specific surgical techniques utilized by the surgeons, patient selection criteria, and clinical notes detailing patient-specific symptoms or workup. While we did attempt to control for different demographic variables in our analysis, a direct match was not performed between vanguard XP and non-Vanguard XP patients. Furthermore, the current threshold for analysis and reporting requires a minimum of 500 cases to be included in the annual MARCQI report. When new implants are introduced to the market, such as the Vanguard XP, there is a period of time required for the registry to collect enough information to comment on overall performance. As such, this limitation in fact potentially highlights the need for future discussion and modification of minimum threshold criteria in order to improve early dissemination of implant performance.

## Conclusions

Arthroplasty implant registries have a a critical role in identifying and reporting implant outcomes. The Vanguard XP experienced higher early failure rates than other TKA implants within the MARCQI registry. The implants usage was focused in certain geographic areas and does not appear in other registry reports. The development of thresholds and benchmarks for registry reporting in collaboration with industry could potentially save patients from the morbidity caused by implants that do not perform as well as anticipated.

## Disclaimer

Although BCBSM and MARCQI work collaboratively, the opinions, beliefs, and viewpoints expressed by the author do not necessarily reflect the opinions, beliefs, and viewpoints of BCBSM or any of its employees.

## Conflicts of interest

Brian R. Hallstrom reports as Michigan Medicine receives partial salary support from Blue Cross Blue Shield of Michigan for my work as Director of MARCQI. Richard E. Hughes reports receiving partial salary support from Blue Cross Blue Shield of Michigan as Senior Advisor of MARCQI. David C Markel reports being a board member/committee appointments for Michigan Orthopedic Society, Michigan Arthroplasty Registry Collaborative Quality Initiative; received royalties, financial or material support from Stryker Orthopedics; has stock or stock options in The CORE Institute, HopCo, Arboretum Ventures; is a paid consultant for Stryker and Smith and Nephew; received royalties from Stryker; and is a paid employee for The CORE Institute. Nicholas B Frisch reports being a board member/committee appointments for AAHKS Advocacy Committee and AAOS OrthoPAC Advisory Board; is a part of medical/Orthopaedic publications editorial/governing board for JOA; received research support from Zimmer Biomet and Canary Medical; has stock or stock options in Advanced Orthopaedic Specialties; and is a paid consultant and a part of speakers bureau for Zimmer Biomet. Michael Masini reports being a part of Executive Committee, MARCQI; and received research support from, a part of speakers bureau for, and is paid consultant for Stryker Corporation. All other authors declare no potential conflicts of interest.

For full disclosure statements refer to https://doi.org/10.1016/j.artd.2024.101478.

## CRediT authorship contribution statement

**Nicholas B. Frisch:** Writing – review & editing, Writing – original draft, Project administration. **Michael A. Masini:** Writing – review & editing, Investigation, Conceptualization. **Huiyong Zheng:** Investigation, Formal analysis, Data curation. **Richard E. Hughes:** Writing – review & editing, Writing – original draft, Supervision, Investigation, Formal analysis, Data curation, Conceptualization. **Brian R. Hallstrom:** Writing – review & editing, Writing – original draft, Data curation, Conceptualization. **David C. Markel:** Writing – review & editing, Writing – original draft, Supervision, Investigation, Data curation, Conceptualization.

## Appendix A Supplementary data

Conflict of Interest Statement for Masini

Conflict of Interest Statement for Zheng

Conflict of Interest Statement for Frisch

Conflict of Interest Statement for Hallstrom

Conflict of Interest Statement for Hughes

Conflict of Interest Statement for Markel
